# Immunomodulation by Inflammation during Liver and Gastrointestinal Tumorigenesis and Aging

**DOI:** 10.3390/ijms22052238

**Published:** 2021-02-24

**Authors:** Nao Nagai, Yotaro Kudo, Daisuke Aki, Hayato Nakagawa, Koji Taniguchi

**Affiliations:** 1 Department of Microbiology and Immunology, Keio University School of Medicine, 35 Shinanomachi, Shinjuku-ku, Tokyo 160-8582, Japan; nnagai@keio.jp (N.N.); adaisuke61@outlook.jp (D.A.); 2 Department of Gastroenterology, Graduate School of Medicine, The University of Tokyo, 7-3-1 Hongo, Bunkyo-ku, Tokyo 113-8655, Japan; yotaro-tky@umin.ac.jp (Y.K.); hayaton0120@gmail.com (H.N.); 3 Department of Pathology, Faculty of Medicine and Graduate School of Medicine, Hokkaido University, North 15, West 7, Kita-ku, Sapporo, Hokkaido 060-8638, Japan

**Keywords:** inflammation, cancer, immunosuppression, tumor microenvironment, aging

## Abstract

Chronic inflammation is thought to promote tumorigenesis and metastasis by several mechanisms, such as affecting tumor cells directly, establishing a tumor-supporting microenvironment, enhancing tumor angiogenesis, and suppressing antitumor immunity. In this review, we discuss the recent advances in our understanding of how inflammation induces the immunosuppressive tumor microenvironment, such as increasing the level of pro-inflammatory cytokines, chemokines, and immunosuppressive molecules, inducing immune checkpoint molecules and cytotoxic T-cell exhaustion, and accumulating regulatory T (Treg) cells and myeloid-derived suppressor cells (MDSCs). The suppression of antitumor immunity by inflammation is especially examined in the liver and colorectal cancer. In addition, chronic inflammation is induced during aging and causes age-related diseases, including cancer, by affecting immunity. Therefore, we also discuss the age-related diseases regulated by inflammation, especially in the liver and colon.

## 1. Introduction

Inflammation is divided into two types by duration, acute inflammation, and chronic inflammation. Acute inflammation is considered beneficial because of its roles in eliminating pathogens and necrotic cells and inducing tissue regeneration and repair [1]. In acute inflammation, immunosuppression occurs after tissue repair or the elimination of pathogens and necrotic cells to resolve inflammation. However, in chronic inflammation, immunosuppression permanently persists due to the lack of the normal negative feedback mechanism.

Chronic inflammation plays a vital role in tumorigenesis and metastasis. Growth factors and pro-inflammatory cytokines induced by chronic inflammation directly enhance cancer cell proliferation, suppress cell death, and induce angiogenesis. Meanwhile, chemokines produced in the tumor microenvironment recruit several types of immunosuppressive cells, including regulatory T (Treg) cells and myeloid-derived suppressor cells (MDSCs), and create an immunosuppressive environment, resulting in tumor initiation and progression. Prostaglandins, essential in inducing inflammatory responses, are also critical mediators in establishing the immunosuppressive microenvironment. Chronic inflammation is also closely related to aging and promotes age-related diseases by affecting immunity.

In this review, we will discuss the recent advances in the research on how inflammation affects antitumor immunity and causes age-related diseases, especially in the liver and colon.

## 2. Inflammation Suppresses Antitumor Immunity

Tumor-promoting inflammation is one of the recognized hallmarks of cancer [2]. Cancer-related inflammation is categorized into four types, which include 1. Chronic inflammation caused by infection and autoimmunity, 2. inflammation caused by environmental and dietary exposure, 3. inflammation caused by therapy-induced exposure, and 4. tumor-associated inflammation [3]. Inflammation, especially chronic inflammation, promotes tumorigenesis and metastasis through several mechanisms, including the suppression of antitumor immunity. Various molecules, cells, and processes, including immune checkpoint molecules, Treg cells and MDSCs, and angiogenesis, are involved in suppressing antitumor immunity mediated by inflammation.

The induction of immune checkpoint molecules in CD8^+^ T cells is crucial in establishing the immunosuppressive microenvironment [4]. The activation of immune checkpoint pathways in CD8^+^ T cells induces T cell exhaustion or dysfunction, resulting in decreased cell proliferation, the expression of effector molecules, such as interferon (IFN)-γ, tumor necrosis factor (TNF), and interleukin-2 (IL-2), immunosuppression, and immunotolerance. Therefore, immune checkpoint inhibitors can be used to treat cancer. The successful use of immune checkpoint inhibitors and chimeric antigen receptor-engineered T (CAR-T) therapy has dramatically changed the significance of immunotherapy in cancer treatment. However, the efficacy of immune checkpoint inhibitors remains unsatisfactory in some cancer types, including colorectal cancer (CRC) [4]. While various combination therapies with potentially improved efficacy are in clinical testing, it remains vital to understand why immune checkpoint inhibitors can only reactivate antitumor immunity in the immunosuppressive microenvironment in a portion of patients.

The limited efficacy of the immune checkpoint inhibitors is due to T cell exhaustion in the tumor microenvironment; therefore, it is crucial to delineate the mechanism of T cell exhaustion. The surface markers for exhausted or dysfunctional T cells are primarily inhibitory receptors, including PD-1, CTLA-4, TIM-3, LAG-3, and TIGIT [5,6,7,8]. The PD-1/PD-L1 axis likely plays the most crucial role in T cell exhaustion in mouse models and clinical outcomes. In addition, compared to the functional effector or memory T cells [9], the transcriptional factors encoded by exhausted or dysfunctional T cells are considerably different, including NR4A (NR4A1, NR4A2, NR4A3), TOX, Eomes, T-bet, Prdm1 (Blimp-1), NF-AT, and BATF [10,11,12,13,14,15]; all these proteins regulate the level of PD-1 [16]. NR4A1 increases the level of PD-1 and TIM-3, and NR4A1-deficient T cells produce more IFN-γ and TNF and mediate tumor regression more significantly. These data suggest that NR4A is an attractive novel target of cancer immunotherapy; for example, camptothecin, a topoisomerase inhibitor, is identified as an NR4A inhibitor and to enhance antitumor immunity [17].

Tregs and MDSCs are also crucial immune cells that induce the immunosuppressive microenvironment [16]. Tregs, which express an essential transcriptional factor, forkhead box P3 (FOXP3), suppress antitumor immunity by producing anti-inflammatory cytokines, such as transforming growth factor (TGF)-β and IL-10 [16]. Furthermore, Tregs repress cytotoxic lymphocytes that produce granzyme and perforin, suppress cell proliferation via IL-2 signaling, and interact with dendritic cells that regulate effector cell function and maturation [18]. CCL17 and CCL22, induced in an inflammatory condition, play an essential role in recruiting Tregs to the tumor microenvironment [19,20]. Once exposed to inflammatory conditions, Treg cells acquire a robust, long-lasting immunosuppressive function [21].

There may be two populations of functionally distinct Treg cells with differential levels of FOXP3. While the Treg population is increased in patients with esophageal cancer, gastric cancer, or CRC and the number of infiltrating Treg cells in a tumor is associated with poor prognosis in most cancer types, the infiltration of FOXP3-positive T cells in CRC is associated with a more favorable prognosis [22]. Therefore, there may be two functionally distinct populations of FOXP3-positive CD4^+^ T cells in CRC, the suppression-competent FOXP3^high^ Treg cells, and the non-suppressive FOXP3^low^ T cells that prevent tumor formation via antitumor immunity [23]. While Tregs are immunosuppressive in inflammatory bowel disease (IBD) and gastritis, they are converted by chronic inflammation to IL-17-producing Tregs, which do not produce IL-10 and are found in colon adenomas [24].

While MDSCs can induce Tregs, they can also suppress effector T cells [25]. MDSCs are a heterogenic population of immature myeloid cells, including progenitors of macrophages, DCs, and granulocytes. MDSCs induce immunosuppression by producing TGF-β, IL-10, reactive oxygen species (ROS), nitric oxide (NO), arginase1, and indoleamine oxidase. MDSCs also promote tumorigenesis by protecting tumor cells from senescence, inhibiting CD8^+^ T cell and natural killer (NK) cell cytotoxicity, and promoting cancer stem cell expansion [26]. The expression of inflammatory mediators, such as IL-1β, IL-6, prostaglandin E_2_ (PGE_2_), granulocyte macrophage colony-stimulating factor (GM-CSF), and vascular endothelial growth factor (VEGF), is increased in the tumor microenvironment, thus recruiting MDSCs in the plasma to the site of inflammation and inducing their activation. In addition, the CXCL8-CXCR2 axis plays a vital role in the recruitment of MDSCs [27].

## 3. Tumor Angiogenesis Develops a Tolerogenic Tumor Microenvironment

Abnormalities in blood vessels also help shape an immunosuppressive microenvironment that induces cancer cells to become aggressive and enable them to resist cytotoxic therapy. Angiogenesis is the development of new blood vessels. Sustained angiogenesis is one of the hallmarks of malignancies since the blood supply is essential for tumor development and growth [28]. Unlike normal blood vessels, the vessels in sustained angiogenesis are structurally and functionally abnormal, immature with abnormally high leakiness [29], and lack pericytes and basement membrane [30]. Additionally, in the tumor microenvironment, they increase the permeability coupled with the elevation in interstitial fluid pressure [30,31]. Angiogenic factors, including VEGF and angiopoietin family proteins, are produced by cancer cells and stromal cells, such as the endothelial and immune cells. VEGF also causes immune suppression by inhibiting the maturation of DCs from progenitors [32], attracting MDSCs in the bone marrow to the periphery [33,34], inhibiting cytotoxic CD8^+^ T cells by inducing the expression of TOX, and upregulating production of PD-1 and other inhibitory checkpoints molecules [35,36]. In addition, VEGF directly induces Treg proliferation in murine models [37]. Collectively, tumor angiogenesis and the tumor’s abnormal vascular structure play critical roles in developing a tolerogenic tumor microenvironment.

Recently, epigenetic dysregulation, which plays a role in tumor angiogenesis and immune evasion, has been proposed as a novel hallmark of cancer [38]. MicroRNAs are also critically important players in remodeling the tumor microenvironment by affecting endothelial cells and several immune cells [39]. However, further studies are necessary to delineate the role of epigenetic dysregulation and microRNAs in the remodeling of the tumor microenvironment.

Next, we will discuss the immunosuppression in the liver and CRC, two cancers significantly driven by inflammation.

## 4. Inflammation and Antitumor Immunity in Hepatocellular Carcinoma

With 745,000 deaths annually, liver cancer is the second biggest cause of cancer deaths worldwide [40]. Hepatocellular carcinoma (HCC) is the predominant form of primary liver cancer. It has several known risk factors, such as chronic hepatitis virus infections, alcohol abuse, autoimmune hepatitis, and several metabolic diseases, including diabetes mellitus [41]. HCC is driven by chronic inflammation [42]. The underlying epidemiology of chronic liver diseases has shifted from being caused by the hepatitis B virus (HBV) or hepatitis C virus (HCV) infection to metabolic disorders, such as non-alcoholic steatohepatitis (NASH) [43]. Regardless of the etiology, most HCCs have underlying cirrhosis caused by chronic liver inflammation [44,45].

Chronic liver inflammation damages hepatocytes, resulting in cell death. This cell death induces compensatory cell proliferation in the liver, a highly regenerative organ. Meanwhile, the inflammation-induced ROS cause DNA damage and consequently increase the frequency of oncogenic mutations. Thus, the compensatory cell proliferation coupled with the accumulated oncogenic mutations induces malignant transformations in the liver [46,47]. Additionally, chronic inflammation supports tumor development and progression by altering the liver immune environments with several mechanisms, allowing malignant cells to escape from immune surveillance.

**Figure 1:**
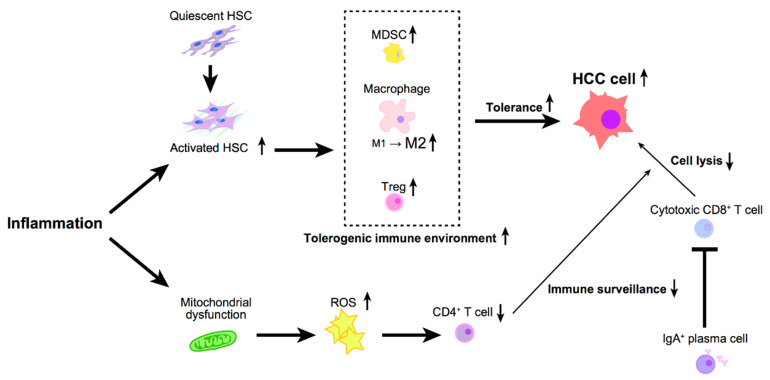
Inflammation oriented cancer-associated immune environment in hepatocellular carcinoma (HCC).

The immune system eradicates emerging cancer cells. The innate immune cells, including macrophages, DCs, and NK cells, are involved in the first line of defense, i.e., monitoring the pathogens and tumor cells. On the other hand, adaptive immunity also contributes to antitumor immunity via the immune surveillance by the CD4^+^ T cells and CD8^+^ T cells (Figure 1).

The activation of innate immune receptors, such as Toll-like receptors (TLRs), via IL-6 induction through a universal TLRs adaptor, MyD88, plays a considerable role in tumor development in the liver [48,49]. Because approximately 75% of the hepatic blood is supplied through the portal vein, the liver is exposed to the potentially immunogenic dietary and microbial products from the gut [50], such as lipopolysaccharide (LPS), a gram-negative bacterial cell component; LPS is also a ligand for TLR4. TLR4 plays a crucial role in hepatocarcinogenesis. TLR4 deficiency ameliorated HCC’s burden in several HCC mouse models, including the diethylnitrosamine (DEN) and carbon tetrachloride (CCl_4_) induction model, or the mice with knocked-out genes encoding the hepatocyte-specific transforming growth factor-β-activated kinase 1 (*TAK1*), phosphatase, or the tensin homolog (*PTEN*) [51,52,53]. The importance of gut-derived LPS has been well studied. LPS depletion by oral antibiotics administration decreased tumor burden in these HCC mice models, while continuous LPS administration enhanced hepatocarcinogenesis [51,53]. Collectively, these data strongly suggest that TLR4 and gut-derived LPS play a critical role in HCC development.

On the other hand, the repression of adaptive immunity also plays a role in HCC development. The number of intrahepatic CD4^+^ T cells is reduced in livers with non-alcoholic fatty liver disease (NAFLD), accelerating hepatocarcinogenesis [54]. Fatty acid accumulation in the NAFLD liver induced the production of mitochondrial ROS, promoting the loss of CD4^+^ T cells and impairing immune surveillance. Moreover, the role of immunoglobulin A (IgA)-producing plasma cells in NASH-associated HCC development was recently reported [55]. There was an accumulation of IgA+ cells in the liver of mice with NASH-induced HCC. These IgA+ cells produce PD-L1 and IL-10 to suppress antitumor cytotoxic CD8^+^ T cells, thus promoting HCC. Genetic or pharmacological interference with IgA+ cells generation attenuated hepatocarcinogenesis, achieving cytotoxic T cell-mediated HCC regression. These data suggest that the inflammation-induced suppression of cytotoxic CD8^+^ T cells was achieved through IgA+ plasma cells, leading to the development of HCC.

Hepatic stellate cells (HSCs) significantly modulate the liver’s immune microenvironment in collaboration with immune cells, despite their limited capacity as antigen-presenting cells (APCs) [56] (Figure 1). HSCs are extracellular matrix-producing stromal cells involved in liver fibrosis; the activation of HSCs promotes HCC development [57,58]. In addition, HSCs change macrophage polarization from an inflammatory (M1) to an immunosuppressive (M2) signature, promoting tumor progression [59,60].

Besides, HSCs contribute to the escape of HCC cells from immune surveillance by regulating the population of Treg cells and MDSCs. HSCs inhibit lymphocyte infiltration in tumors, induce apoptosis of infiltrating mononuclear cells, and enhance the expression of PD-L1 and the number of CD4^+^CD25^+^ Treg cells in immunocompetent mice [57]. HSCs store retinoids to produce retinoic acid during activation. It has been shown that isolated murine HSCs enhance the differentiation of isolated naive OT-II T cells co-cultured with splenic DCs in the presence of TGF-β1 into functional Foxp3+ Treg cells in an all-trans-retinoic acid-dependent manner. This study indicates that HSCs influence DC-primed T cells to differentiate into functional Treg cells, resulting in the liver’s tolerogenicity [61].

The capacity of HSCs to transform peripheral blood monocytes into MDSCs has been studied [62]. Isolated human monocytes from healthy donors co-cultured with human HSCs display downregulation of HLA-DR and develop a phenotype similar to that of MDSCs. This induction of MDSCs is only achieved upon co-culture with activated HSCs but not with quiescent HSCs, and is mediated by the cells’ physical interaction via CD44 but not by soluble factors.

HSCs also suppress immunity by inhibiting cytotoxic CD8^+^ T cell activation via a CD54-mediated mechanism [63]. Isolated murine HSCs or human HSC cell line LX-2 prevent the priming of naive CD8^+^ T cells by APCs. The inhibition of T cell activation is associated with HSC activation. Mechanistically, the increased level of CD54 on HSCs restricts the production of IL-2 receptor and IL-2 in T cells, which attenuates T cell activation.

Most of the tolerogenic mechanisms of HSCs depend on the activated state of HSCs. HSCs are activated in the inflamed liver via several mechanisms. For example, inflammation activates caspase-1 and increases the release of IL-1β, which facilitates parenchymal liver injury. Furthermore, danger-associated molecular patterns (DAMPs) released from dying hepatocytes promote HSCs activation via inflammasome signaling; NACHT, LRR, and PYD domains-containing protein 3 (NLRP3), a significant component of the inflammasome and a downstream of DAMPs, contribute to HSC activation [64]. In addition, IL-33, which is released from damaged hepatocytes, also contributes to HSC activation through the expansion of innate lymphoid cells [65]. Moreover, hepatic macrophages, recruited in the damaged liver, produce cytokines and chemokines, including TGF-β, PDGF, TNF, IL-1β, CCL3, and CCL5, which lead to HSC activation [66,67,68,69]. As mentioned above, HSCs are activated during liver inflammation; inflammation plays a crucial role in immune suppression via HSCs activation.

## 5. Inflammation-Induced Immunoregulation in Colorectal Cancer

**Figure 2:**
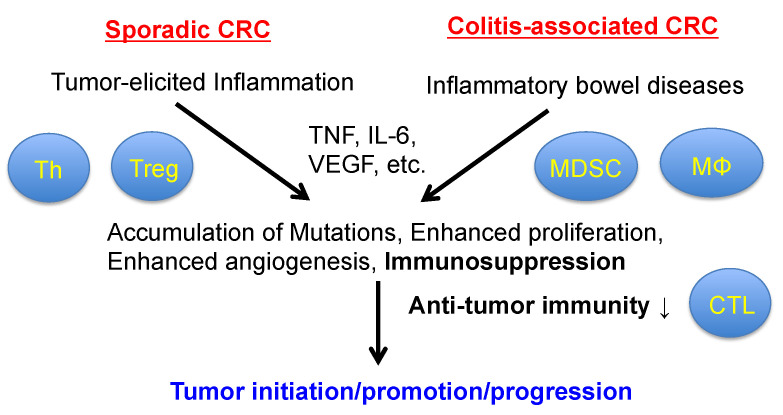
The role of inflammation in colorectal tumorigenesis.

Chronic inflammation is associated with CRC, especially IBD-induced CRC. Chronic inflammation even plays a crucial role in the development and progression of sporadic CRC, also known as “tumor-elicited inflammation”, by inducing the loss of the tumor suppressor adenomatous polyposis coli (APC) [70,71,72] (Figure 2). The loss of APC in colonic epithelial cells induces adenoma formation through β-catenin activation, resulting in a decrease in the mucus layer and barrier dysfunction [70]. Consequently, gut bacteria or bacterial products or both, such as LPS, translocate into the lamina propria and activate the TAMs. The TAMs produce pro-inflammatory cytokines, including IL-23, which activates IL-17-producing cells, such as Th17 cells. Finally, pro-inflammatory cytokines, including TNF, IL-6, and IL-17 produced by IL-17-producing cells, directly promote the proliferation and survival of adenoma cells via the activation of NF-κΒ and STAT3 [70,71]. TNF and IL-6 are two major pro-inflammatory cytokines produced at high levels in human CRC; they mainly activate the IKK-NF-κB and JAK-STAT3 pathways, respectively [73,74]. IL-6 also activates the Ras-MAPK, PI3K-Akt, and Src family kinase (SFK)-YAP pathways [74,75]. In addition to activating β-catenin, the loss of APC continuously activates the SFK-YAP pathway by establishing a positive feedback loop through the upregulation of gp130/IL6ST, a common receptor for the IL-6 family cytokines. The upregulation of gp130 on the tumor cell surface also leads to the response to a low concentration of IL-6 in low-grade inflammation [72]. IL-6 suppresses antitumor immunity by several mechanisms [76,77]. In a mouse experiment using the mouse colon cancer cell line CT-26, IL-6 deletion in mice enhances antitumor immunity by augmenting type-1 immunity, suggesting the immunosuppressive role of IL-6 in the tumor microenvironment [78]. On the other hand, it is unclear whether TNF is involved in immunosuppression.

## 6. Induction of Aging-Related Diseases by Inflammation

Aging is a complex and heterogeneous process characterized by the progressive loss of physiological integrity that leads to functional impairment and increased susceptibility to death [79]. Aging is affected by environmental, genetic, and epigenetic factors; it is a significant risk factor for cancer [80]. Recent studies revealed that chronic inflammation functions as a critical driver of aging and age-related diseases [81,82,83,84,85,86]. Inflammaging is one of the hallmarks of aging. It is indicated by the increased levels of circulating TNF, IL-6, and C-reactive protein and defined as an age-related chronic, systemic, and unresolved low-grade inflammation [87,88]. Inflammaging causes cytokine dysregulation and affects the immune system with the progressive activation of macrophages and related cells, potentially linking aging to immunosenescence and age-related diseases, such as cancer, Alzheimer’s disease, rheumatoid arthritis, cardiovascular diseases, and type II diabetes [89].

Various cells and molecules are involved in inflammaging. Although many cell types, including epithelial, fibroblast, and endothelial, are related to inflammation in age-related diseases, immune cells mainly induce chronic inflammation during aging.

It is thought that the continuous upregulation of pro-inflammatory mediators, such as TNF, IL-6, COX-2, and iNOS, is induced during aging due to the imbalance in redox status and oxidative stress that activate pro-inflammatory signaling pathways, including NF-κB, and STAT3 [90,91]. A two to four-fold increase in the serum levels of inflammatory mediators is observed during aging [89]. Additionally, it is reported that coagulation factors activate inflammatory signaling beyond their original role in the coagulation system [82]. Moreover, microRNAs link inflammaging with cellular senescence and cancer [92].

Also, some cellular processes are involved in inflammaging. The upregulation of endoplasmic reticulum (ER) stress as well as the accumulation of inflammasome and lipid are also observed during aging and might play an important role in age-related diseases. Lastly, cellular senescence and dysregulated innate immunity have been found to cause prolonged chronic inflammation even after the initial stimulus has been removed [82].

Aging is accompanied by the change of epigenetic information in both dividing and non-dividing cells. Epigenetic changes significantly influence the aging process, life span, and induction and progression of age-related diseases, including cancer [93,94,95]. Diet plays an essential role in inducing epigenetic changes; however, it is challenging to predict which epigenetic alterations induced by different diets are beneficial or detrimental for aging. Further studies are required.

## 7. Liver Diseases with Aging

Most human malignancies, including HCC, are considered age-related diseases because their incidences increase with age [42,96]. Aging is an independent risk factor, at a hazard ratio of 1.02 per year, for the overall survival of HCC patients with both hepatitis virus-related or non-viral etiologies [97,98].

An increased number of senescent hepatocytes are observed among chronic liver diseases of several etiologies. In the aged liver, the number of senescent cells is also increased in hepatocytes and stromal cells, including stellate and immune cells. Cellular senescence develops due to telomere shortening and accumulated DNA damage mediated by oxidative stress [99]. Morphological changes in hepatocytes in aged livers can be associated with increased polyploidy and mitochondrial dysfunction [100,101], which enhance the liver’s susceptibility to damage and decline in regeneration [102].

Meanwhile, senescent cells also secrete pro-inflammatory cytokines to accelerate the development of chronic liver diseases [103]. In addition, aged hepatic stellate cells demonstrate enhanced activity [104]. The aged liver sinusoidal endothelial cells display dysregulated vasodilatory capacity, which leads to portal hypertension contributing to developing liver cirrhosis and liver dysfunction in chronic liver diseases [104]. On the other hand, the aging of Kupffer cells, the residual liver macrophages, has been reported to be heterogeneous and context-dependent [105].

Considering the importance of chronic inflammation and the dysregulated immune surveillance in the pathogenesis of chronic liver diseases and HCC, it is likely that inflammaging plays a specific role in these liver diseases. There is accumulating evidence demonstrating inflammaging’s contribution to immune dysfunction [106]. However, it remains to be elucidated whether and how inflammaging contributes to the pathogenesis of liver diseases, including HCC.

## 8. Inflammation and Aging in the Gastrointestinal Tract

During aging, somatic clones with driver mutations expand in normal tissues, resulting in cancer development. During inflammaging, the pathophysiological alterations in the colon occur at molecular and cellular levels, and the regenerative ability of the epithelium is decreased by inflammation [86,107]. Recently, two Japanese groups have reported similar findings that such clonal expansion occurs in the inflamed colon epithelium of patients with ulcerative colitis [108,109]. The epithelium accumulates somatic mutations in several genes associated with IL-17 signaling, including *NFKBIZ*, *TRAF3IP2*, *ZC3H12A*, *PIGR*, and *HNRNPF*. These mutations lead to the downregulation of IL-17 and other pro-inflammatory signals. However, these somatic mutations are rarely found in sporadic and colitis-associated CRC, suggesting the negative selection for mutated epithelium during CRC development.

The development of esophageal cancer, especially esophageal squamous cell carcinoma, is highly associated with chronic inflammation induced by tobacco smoking and alcohol drinking and pro-inflammatory cytokines, including the IL-6 family cytokines [74,110]. The clonal expansion of epithelium in the inflamed colon is also observed in the esophageal epithelium during aging [111]. The progressive age-related expansion of the esophageal epithelial clones with driver mutations (predominantly *NOTCH1*) is substantially accelerated by alcohol drinking and heavy smoking. A high frequency of *NOTCH1* and *PPM1D* mutations are observed in the physiologically normal esophageal epithelial cells, compared to the common mutations in esophageal carcinoma. Therefore, the observed remodeling of the esophageal epithelium by driver-mutated clones is an inevitable result of normal aging, leading to esophageal tumorigenesis.

## 9. Immune Signal Modulation as a Potential Therapeutic Target of HCC and CRC

An immunologically active “inflamed” tumor microenvironment has been associated with improved prognosis in HCC patients [112,113,114]. In one instance, the intratumor immune environment of 234 primary HCCs was assessed by examining their cytolytic activity (CYT), defined as the average production of granzyme A and perforin [115], and was found to be immunosuppressive, consistent with the discussion above [116]. The immune gene signatures of these primary HCCs were characterized to categorize the HCCs into four subclasses by different active immunosuppressive mechanisms: Tumor-associated macrophage (TAM), CTNNB1, CYT, and Treg. Interestingly, the Treg subgroup showed poorer prognosis, suggesting that the impact of Treg induction had a pivotal role in shaping the tolerogenic state of the HCCs.

Immune checkpoint molecules are critical in inhibiting antitumor immunity; among them, PD-1 and CTLA-4 have been studied extensively [117]. Therapeutic strategies targeting these molecules aim to block T cell-mediated tumor immunosuppression and Treg activity, consistent with a previous report that emphasizes the significance of Treg activity in a tumor immunosuppressive environment [116].

Clinical trials have been carried out to test monoclonal antibodies targeting immune checkpoint molecules; these therapeutics have been found efficacious in suppressing tumor growth, resulting in favorable patient prognosis. Thus, they have already been approved by the United States Food and Drug Administration (FDA) for unresectable or metastatic HCC [118,119,120] and CRC [121,122] (Table 1 and Table 2). Immune checkpoint inhibitor therapy is only effective in CRC patients who have microsatellite instability-high (MSI-H) or mismatch repair-deficient (dMMR) tumors or both. Recently, pembrolizumab was approved by FDA as a first-line therapy for patients with unresectable or metastatic MSI-H or dMMR CRC.

An animal experiment using the transplantation model of CRC cells demonstrated that a combination of PD-1 antibodies with VEGF inhibition achieved a strong and synergic antitumor effect, consistent with the previous finding that VEGF produced by tumors shapes the immunosuppressive microenvironment. Lenvatinib, a multi-kinase inhibitor with anti-angiogenic potential and a clinically used agent in advanced HCC patients, induced more significant tumor regression combined with the PD-1 antibodies in the HCC mouse model [123]. Indeed, an emerging regimen involving a PD-L1 monoclonal antibody, atezolizumab, and the VEGF inhibitor bevacizumab has recently received FDA approval for its demonstrated improvement in the overall and progression-free survival in patients with unresectable HCC compared to that of the approved first-line treatment with a multi-kinase inhibitor, sorafenib [124].

Also, non-steroidal anti-inflammatory drugs (NSAIDs) are shown to have both protective and therapeutic effects against cancer [125]. The long-term use of NSAIDs reduces the risk of familial adenomatous polyposis as well as sporadic CRC. In addition, NSAIDs decrease the incident and recurrent risk of HCC. However, the NSAIDs’ mechanisms of suppressing tumorigenesis and metastasis are still controversial.

## 10. Conclusions and Perspective

Chronic inflammation, which is closely related to establishing the immunosuppressive tumor microenvironment, dampens the effect of cancer immunotherapeutics, such as immune checkpoint inhibitors and CAR-T cells. Therefore, it is vital to develop a method to shift the immunosuppressive tumor microenvironment to an immunocompetent state. IL-6 might be one of the attractive targets for improving the efficacy of cancer immunotherapy. It might also delay aging and alleviate age-related diseases by modulating the pro-inflammatory environment and subsequently resolving inflammation.

## Figures and Tables

**Table 1:** FDA-approved checkpoint molecules in HCC.

Drug	Target	Line of Therapy	Administration
Atezolizumab	Fully human, anti-PD-L1 IgG1 monoclonal antibody	1st	Used in combination with bevacizumab for patients with unresectable HCC
Nivolumab	Fully human, anti-PD-1 IgG4 monoclonal antibody	2nd	Used alone or with ipilimumab for patients who have already been treated with sorafenib
Pembrolizumab	Fully human, anti-PD-1 IgG4 monoclonal antibody	2nd	Used for patients who have already been treated with sorafenib
Ipilimumab	Fully human anti-CTLA-4 IgG1 monoclonal antibody	2nd	Used in combination with nivolumab for patients who already been treated with sorafenib

**Table 2:** FDA-approved checkpoint molecules in CRC.

Drug	Target	Line of Therapy	Administration
Pembrolizumab	Fully human, anti-PD-1 IgG4 monoclonal antibody	1st	Used alone for patients with unresectable or metastatic MSI-H/dMMR CRC
Nivolumab	Fully human, anti-PD-1 IgG4 monoclonal antibody	2nd	Used in combination with ipilimumab for patients with metastatic MSI-H/dMMR CRC that progressed following treatment with a fluoropyrimidine, oxaliplatin, and irinotecan
Ipilimumab	Fully human anti-CTLA-4 IgG1 monoclonal antibody	2nd	Used in combination with nivolumab for patients with metastatic MSI-H/dMMR CRC that progressed following treatment with a fluoropyrimidine, oxaliplatin, and irinotecan
